# Manipulation Therapy Prior to Diagnosis Induced Primary Osteosarcoma Metastasis—From Clinical to Basic Research

**DOI:** 10.1371/journal.pone.0096571

**Published:** 2014-05-07

**Authors:** Jir-You Wang, Po-Kuei Wu, Paul Chih-Hsueh Chen, Chuen-Chuan Yen, Giun-Yi Hung, Cheng-Fong Chen, Shih-Chieh Hung, Shih-Fen Tsai, Chien-Lin Liu, Tain-Hsiung Chen, Wei-Ming Chen

**Affiliations:** 1 Department of Orthopaedics, Taipei Veterans General Hospital, Taipei, Taiwan; 2 Department of Orthopaedics, Therapeutical and Research Center of Musculoskeletal Tumor, Taipei Veterans General Hospital, Taipei, Taiwan; 3 Institute of Traditional Medicine, School of Medicine, National Yang-Ming University, Taipei, Taiwan; 4 Department of Surgery and Institute of Clinical Medicine School, National Yang-Ming University, Taipei, Taiwan; 5 Department of Pathology and Laboratory Medicine, Taipei Veterans General Hospital, Taipei, Taiwan; 6 Division of Hematology and Oncology, Department of Medicine, Taipei Veterans General Hospital, Taipei, Taiwan; 7 Department of Pediatrics, Taipei Veterans General Hospital, Taipei, Taiwan; 8 Institute of Clinical Medicine, National Yang-Ming University, Taipei, Taiwan; 9 Department of Pharmacology, National Yang-Ming University, Taipei, Taiwan; 10 Division of Molecular and Genomic Medicine, National Health Research Institutes, Zhunan, Miaoli, Taiwan; China Medical University, Taiwan

## Abstract

Osteosarcoma (OS) patients who suffer manipulation therapy (MT) prior to diagnosis resulted in poor prognosis with increasing metastasis or recurrence rate. The aim of the study is to establish an *in vivo* model to identify the effects of MT on OS. The enrolled 235 OS patients were followed up in this study. *In vivo* nude mice model with tibia injection of GFP-labeled human OS cells were randomly allocated into MT(+) that with repeated massage on tumor site twice a week and no treatment as MT(−) group. The five-year survival, metastasis and recurrence rates were recorded in clinical subjects. X-ray plainfilm, micro-PET/CT scan, histopathology, serum metalloproteinase 2 (MMP2), metalloproteinase 9 (MMP9) level and human kinase domain insert receptor (KDR) pattern were assayed in mice model. The results showed that patient with MT decreased 5-year survival and higher recurrence or metastasis rate. Compatible with clinical findings, the decreased body weight (30.5±0.65 g) and an increased tumor volume (8.3±1.18 mm^3^) in MT(+) mice were observed. The increasing signal intensity over lymph node region of hind limb by micro-PET/CT and the tumor cells were detected in lung and bilateral lymph nodes only in MT(+) group. MMP2 (214±9.8 ng/ml) and MMP9 (25.5±1.81 ng/ml) were higher in MT(+) group than in MT(−) group (165±7.8 ng/ml and 16.9±1.40 ng/ml, individually) as well as KDR expression. Taking clinical observations and *in vivo* evidence together, MT treatment leads to poor prognosis of primary osteosarcoma; physicians should pay more attention on patients who seek MT before diagnosis.

## Introduction

Osteosarcoma (OS) is a common primary malignant tumor occurring in childhood or adolescence[1] which is frequently located on the parts with rapid bone growth such as distal femur, proximal tibia, or proximal humerus[2], [3]. OS has high local aggressiveness and occasionally metastasis to lung and other bone sites, with few occasions to lymphoid metastasis[4]. Recently, the combination of practicable surgery and chemotherapy improve the long-term survival of approximately 60–70% that prolong the survival rate of OS[5]. Nonetheless, issue such as complementary treatments before OS diagnosis affecting the clinical outcome remains important but less discussed.

Because of the young age and the most common symptoms of OS are pain and swelling, many patients seek for manipulative therapy (MT), such as massage, Tuina, or other kinds of complimentary treatments prior to diagnosis to release these uncomfortable symptoms, especially in Asian countries[6]. The full-body massage therapy also known and applied for release tumor metastasis-induced bone pain on cancer patients[7]. However, though such therapies may improve or release such symptoms, there are still some questions about such kinds of treatments on tumor.

It was been reported from 1922 by Knox that massage on cancer patients may showed some risk to induce metastasis[8]. In 2004, Diaz et al provided the evidence that mechanical treatments induced distal metastasis by lymphoid leaking[9]. Our previous clinical study has demonstrated that patients receiving MT prior to diagnosis of OS shows lower survival rate accompanied with higher local recurrence and lung metastasis rate[6]. However, the mechanisms of MT-induced metastasis of OS remains further elucidated.

In this study, by using x-ray diagnosis, micro-PET/CT image, histopathology and serum MMP9 level, we established an animal OS model and provide in vivo evidence of MT-induced metastasis of osteosarcoma from clinical to basic research.

## Materials and Methods

### Subjects

The human study was proved by ethics committee of Taipei Veteran's General Hospital (IRB No. 2011-05-005IC). A total of 235 patients with osteosarcoma were included in this study from July 1995 to June 2012. The patients who had axially-located tumors (n = 6), low-grade malignancies (n = 13), prior surgical treatment at another facility (n = 16) were excluded from the study. In addition, 1 patient was lost to follow-up. Therefore, a total of 200 patients were ultimately included in the study. Of these 200 patients, 104 patients had received MT prior to diagnosis and 96 patients had not received MT. Each patient gave written informed consent. Since the formulas of chemotherapy for OS patients are various in different hospitals and may cause variety prognosis. To avoid the confounding factor of different chemotherapy, subjects enrolled in this study were under the same formula of chemotherapy and patients who received other chemo-treatment in other hospital were excluded in this study.

### Cells and culture condition

MG-63 (CRL-1427) was purchased from American Tissue Culture Collection (ATCC, Rockville, MD) and cultured in high-glucose Dulbecco's modified Eagles medium (HG-DMEM) with 10% FBS and for passaging, cells were digested by 0.25% trypsin (GIBCO-BRL, Gaithersburg, MD) followed by suspended with culture medium. MG-63 with GFP reporter was established by transfection of GFP-lentivirus to MG-63cells. Briefly, GFP-lentivirus was purchased from National Science Council RNAi core facility, Academia Sinica Taiwan. Subconfluent MG-63 cells were infected with GFP-lentivirus in the presence of 8 ìg/mL polybrene (Sigma, St. Louis, Mo). At 24 h post-infection, media were removed and replaced with fresh growth media containing puromycin (1 ìg/mL) to select for infected cells[10], [11].

### *In vivo* tumor xenograft and manipulation model

**Figure 1:**
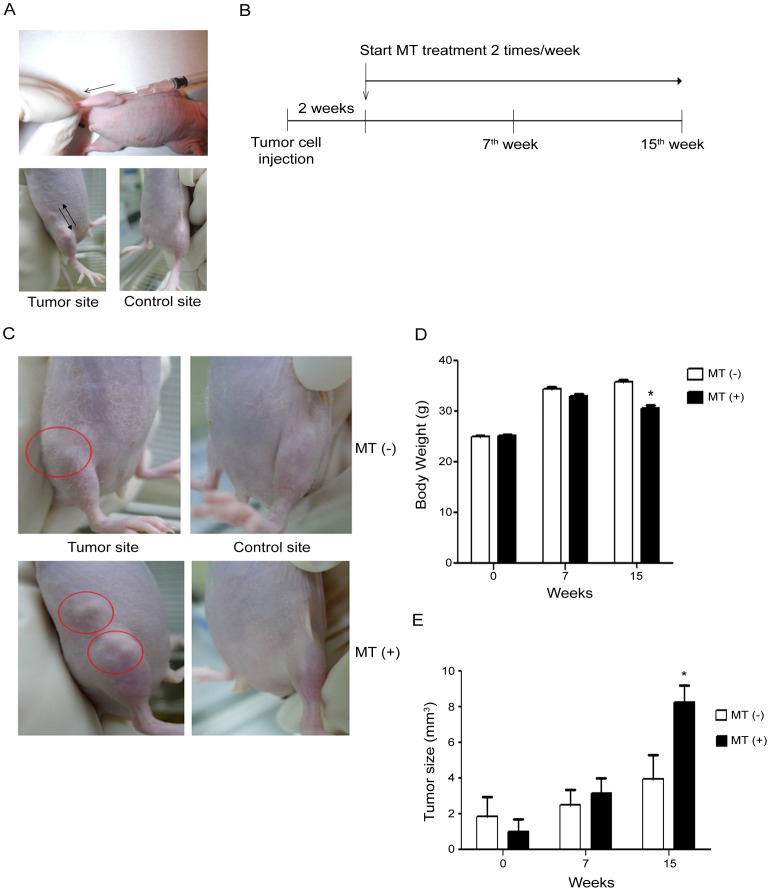
MT treatment in the *in vivo* OS animal model decreased body weight and increased tumor size. Human OS MG63 cells previously-labeled with GFP were injected into right tibia with the number of 1×10^7^/ 0.05 ml in each marine to perform OS mice model while no treatment in left tibia as normal control (A). About 2wk injection with the tumors being noticed in right tibia, MT treatment was performed twice a week for 7 or 15 wk as the experiment protocol (B). Apparently, enlarged mass and inguinal lymph nodes were noted in MT (+) group at bilateral tibia regions but not in MT (−) group (C). Though all of the mice formed tumor in week 15 (D), the decreased body weight accompanied with the increased tumor size were noticed in MT (+) group. The results showed that MT (+) group showed decreased body weight (E, 30.5±0.65 g, ▪) and increased tumor volume (F, 8.3±1.18 mm^3^, ▪) compared to MT (−) group with body weight (E, 35.8±0.40 g, □) and tumor volume (F, 3.9±1.34 mm^3^, □), respectively. (*, p<0.05 compare with MT (+) versus MT (−) groups).

Study protocols involving mice were approved by the Institutional Animal Committee of Taipei Veterans General Hospital (IACUC 2012–188) under the animal welfare and steps. Immunodeficient NU-Foxn1nu mice were obtained from The LASCO Laboratory and maintained as a clone at the Taipei Veterans General Hospital Animal Facility (Taipei, Taiwan) in specific pathogen-free conditions. The mice were used for experiments at 8 weeks of age. MG63 human OS cells were infected by GFP-lentivirus and GFP-labeled OS cells were injected into right tibia from knee joint for 1×10^7^ cells/0.1 ml PBS for each mouse, leading to a solid tumor noticeable around the injection site at day 14, while the left site of the same mouse as used as a negative control[12], [13]. After tumor formation, mice were randomly allocated into two groups, namely MT (+, n = 15) and MT (−, n = 15) groups. For MT (+) mice, MT was conducted with massage on tumor site for five cycles back and forth each time and twice a week for 7 or 15 weeks as described previously (Fig. 1B)[14], [15]. The mice were sacrificed when the tumor size was almost 2% of body weight and collected tissues including bilateral legs, liver, lung, bilateral inguinal lymph nodes, and serum for immunohistochemistry and MMP9 protein analysis.

### X-ray image for mice after treatment

The mice were taken X-ray plain film before sacrifice after tumor formation by radiography (AXR minishot 160 KV). The definitions of plain film signatures of OS including osteoblastic and/or osteolytic bone lesion, aggressive periosteal reaction, and visible soft tissue mass. The radiographs were digitized and subsequently analyzed using Adobe Photoshop [16].

### Micro-PET/CT

After MT treatment, Micro-PET/CT for dynamic study was conducted on mice before sacrifice. Briefly, the mice were injected with 0.3 mCi [^18^F]-FDG via tail vein followed by micro-PET R4 scanner (Concorde MicroSystems, Knoxville, TN with energy window being 350–650 keV, timing window being 6 ns). Dynamic sinograms were produced with 12×10 s, 6×30 s, 5×300 s, 3×600 s and 4×900 s frames. The images were reconstructed by the Fourier rebinning algorithm and 2-D filtered back projection using a ramp filter with cutoff at Nyquist. All these processes were carried out by MicroPET Manager (version 2.3.3.6) under the instruction of the manufacturer. The PET images were analyzed using ASIPro VM6.3.3.1 software (Concorde MicroSystems, Knoxville, TN). A cylinder calibration method was used to convert the image units from cps per voxel (cps/voxel) to nCi per cm3 (nCi/cm3). Finally, the CT scan was performed by the same machine[17], [18].

### Immunohistochemistry

After animal sacrificed, bilateral legs, bilateral inguinal lymph nodes, liver, and lung tissues of each animal were paraformaldehyde-fixed, embedded, and sectioned for histopathological examination. The tissue samples were performed with hematoxylin-eosin stain (H&E stain) for histopathology. To confirm the location of GFP (+) tumor cells that had been injected previously, the rabbit anti-GFP antibody (Genetex, USA, 1∶1000) was used for recognizing GFP (+) MG63 cells in the organs of tumor-transferred mice. The expression level of human KDR was detected to identify the angiogenesis pattern in injected tumor under MT positive or negative treatments.

### MMP9 gel-shift assay

To investigate the possible mechanism of MT induced-metastasis, matrix metalloproteinase 9 (MMP9) expression levels in mice serum was detected by gel-shift zymography assay. Briefly, 500 µl serum was centrifuged by centrifugal filter units as 9000 rpm for 30 minutes and followed by electrophoresis. The gel was stained by Coomassie blue for 20 min.

### ELISA assay

To quantitate the serum level of MMP2 and MMP9, human ELISA assay kit (R&D System, USA) were performed followed by the manufacture's protocol.

### Statistical method

The data were expressed as mean ± standard deviation (SD). Patients and in vivo tumor formation data were analyzed by Chi square analysis. Independent t test was performed for comparison of data of independent samples. More than two groups were compared by one-way ANOVA. A p value <0.05 was considered statistically significant.

## Results

### Prior MT decreased the survival rate and increase metastasis risk clinically in OS patients

The enrolled 235 osteosarcoma patients from 1995–2012 with the sign up of patient inform concern. 200 cases were included and 104 cases received MT prior to diagnosis while 96 cases did not (Table 1). The gender (p = 1) and average age (p = 0.87) between MT and NMT group showed no significant difference. The five-year survival rate was significant decreased in MT group (52.9%) compare to NMT group (97.9%, p<0.0001). Though the tumor volume between these two group revealed no significant change (276±43.9 mm^3^ in NMT and 285±39.5 mm^3^ in MT group, p = 0.87), the recurrence rate (28.8%) and lung metastasis rate (38.5%) in MT group were higher than NMT group (6.25%, p<0.0001 and 8.3%, p<0.0001, individually). With no significant skip lesion in NMT group, the skip lesion rate in MT group was up to 11.5% (p = 0.0004). From patients follow up, MT treatment prior to diagnosis induced poor prognosis of primary OS.

**Table 1:** Patient profiles and osteosarcoma characteristics.

	non-manipulation (NMT)	manipulation (MT)	p value
	(n = 96)	(n = 104)	
Gender			1
male	66	72	
female	30	32	
average age	21.7±1.55	18.3±1.10	0.0738
**Recurrence rate** *	**6.25%**	**28.8%**	**<0.0001**
non-recurrence	90	74	
recurrence	6	30	
**lung metastasis** *	**8.3%**	**38.5%**	**<0.0001**
no meta	88	64	
meta	8	40	
**skip leision** *	**0%**	**11.5%**	**0.0004**
no skip leision	96	92	
skip leision	0	12	
**tumor volumn (mm∧3)**	**276±43.9**	**285±39.5**	**0.87**
**5-year survival** *	**94 (97.9%)**	**55 (52.9%)**	**<0.0001**

*: P<0.05 by Chi square test.

### Intra-tibia injection of MG63 formed tumor on Nude mice while MT induced tumor growth and decreased body weight

Human OS MG63 cells previously-labeled with GFP were injected into right tibia sites in each mice to perform OS mice model, while no treatment in left tibia as normal control (Fig. 1A, upper panel). About 2 wk injection with the tumors being noticed in right tibia, MT treatment was performed on tumor site twice a week for 7 or 15 wk (Fig. 1A, lower panel, Fig. 1B). Apparently, noted mass over tibia and inguinal area were noted in 2 weeks after tumor cells injection while the tumor volume was enlarged in MT (+) group (Fig. 1C) but not in MT (−) group. Though in different progression rate, each mice that been injected by MG63 cells formed tumor mass during 15-weeks experiment period. Total body weight and tumor volume in each group was also measured at 7^th^ and 15^th^ wk after MT treatment. The results showed that MT (+) mice showed a decreased body weight (30.5±0.65 g) and an increased tumor volume (8.3±1.18 mm^3^) compared to MT (−) group with body weight (35.8±0.40 g) and tumor volume (3.9±1.34 mm^3^), respectively. (Fig. 1D, p<0.001; Fig. 1E, p = 0.038) The results indicated that MT treatment induced tumor growth and decreased the body weight in xenograft mice model.

### Radiography image of mouse OS model by X-ray plain film

**Figure 2:**
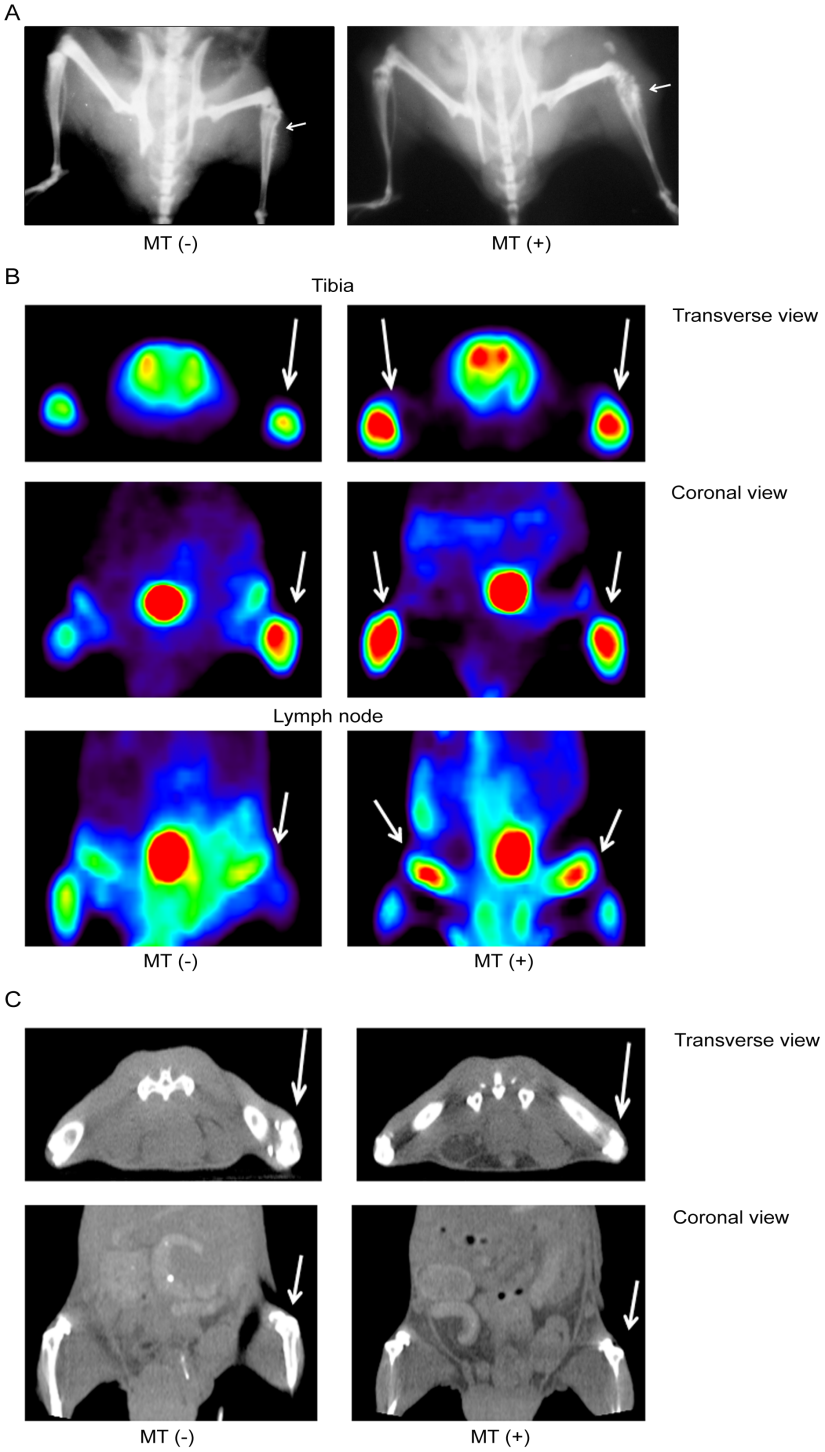
The X-ray radiography and the Micro-PET/CT demonstrated the significant signal in tibia and lymph nodes in MT (+) group. X-ray radiography was taken in the mice for monitoring the formation of OS 7^th^ or 15^th^ wk after tumor injection. The sunburst features and bone lesions were noticed in right tibia in MT (−) group (A, left) and MT (+) group (A, right). The white arrow heads pointed to the sunburst sites. Micro-PET/CT scan were performed for image detection of tumor migration. The enhanced signal on right tibia of MT (−) group (B, left panel) and bilateral tibia and lymph node of MT (+) group (B, right panel) were detected by micro-PET and micro-CT scanning (C, arrows means tumor injection site).

X-ray radiography was taken in the mice for monitoring the formation of OS in 7^th^ or 15^th^ wk after tumor injection. The signatures of sunburst and bone lesion were noticed on tumor injection site on right tibia in both MT (−) (Fig. 2A, left) and MT (+) group (Fig. 2A, right). Micro-PET/CT scan were performed for image detection of tumor migration. The enhanced signal on right tibia of MT (−) group (Fig. 2B, left panel) and bilateral tibia and inguinal lymph node of MT (+) group were detected by micro-PET (Fig. 2B, right panel) and micro-CT scanning (Fig. 2C). The image results indicated the tumor cell migration under MT treatment.

### The injected tumor cells were detected in lung after 15 weeks MT treatment by histopathology stain

**Figure 3:**
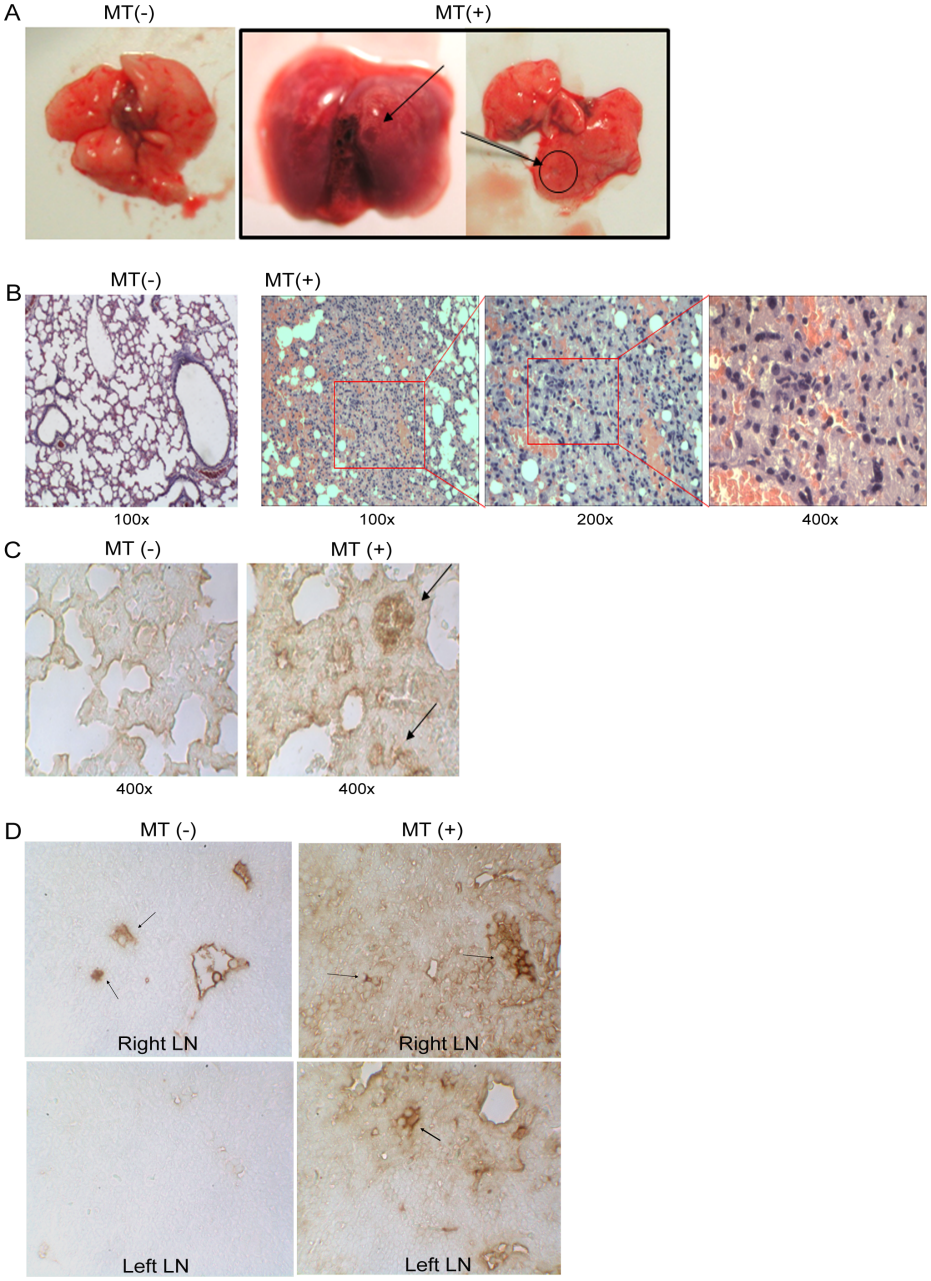
The histopathology of MT (+) group showed cell migration into lung and lymph nodes. After MT (+) or MT (−) treatment for 15 wk, the mice were sacrificed and the lung tissues were examined by histopathology with H&E stain. The tumor nodules were noticed in lung lobes in MT (+) group (A) and the alveoli were filled with tumor cells in MT (+) group, but not in MT (−) group (B). To provide direct evidence that MT induces metastasis in primary OS model, we labeled MG63 with GFP reporter gene and traced the GFP (+) tumor cells by immunohistochemistry. The GFP (+) cells were detected in lung nodules in MT (+) group while was undetectable in MT (−) group (C). In the lymph nodes, the GFP (+) cells were detected in right side where the tumor cells were injected in both MT (+) and MT (−), while in MT (+) group, the GFP (+) tumor cells were detected in bilateral lymph nodes (D).

In 15-mice of each group, The metastasis rate in MT (+) was significant higher than MT (−) group (40% versus 0%, P = 0.00169, Table 2). Though the lymph node lesion was not significant different, it was higher in MT (+) than in MT (−) group (53.3% versus 13.3%, P = 0.0502, Table 2). To tracking the mobility of tumor cells after MT (+) or MT (−) treatment for 15 wk, the injected MG63 cells were labeled with GFP for H&E stain and anti-GFP immunohistochemistry stain were performed for histopathology. The tumor nodules were noticed in lung lobes in MT (+) group (Fig. 3A) and the alveoli were filled with tumor cells in MT (+) group, but not in MT (−) group (Fig. 3B). GFP(+) tumor cells were also detected in lung (Fig. 3C) and bilateral lymph node (LN) (Fig. 3D, right panel) in MT (+) group. While few GFP(+) cells were noted in the LN of tumor injected site of MT (−) group, the lung and contralateral LN (Fig. 3D, left panel). The lung metastasis rate in MT (+) was significantly higher than MT (−) group (40% versus 0%, Table 2). The results showed that tumor cells migration into lung tissue in MT-treated mice.

**Table 2:** The lung and LN metastasis under MT treatment in mice model.

	Lung metastasis		LN skip lesion	
	MT (−)	MT (+)	MT (−)	MT (+)
W0	0/15	0/15	0/15	0/15
W7	0/15	2/15	0/15	4/15
W15	0/15	6/15	2/15	8/15
Metastasis rate	0%	40.0%	13.3%	53.3%
	P = 0.00169*		P = 0.0502	

*: P<0.05 by Chi square test.

### MT treatment increased the serum level of MMP2 and MMP9

**Figure 4:**
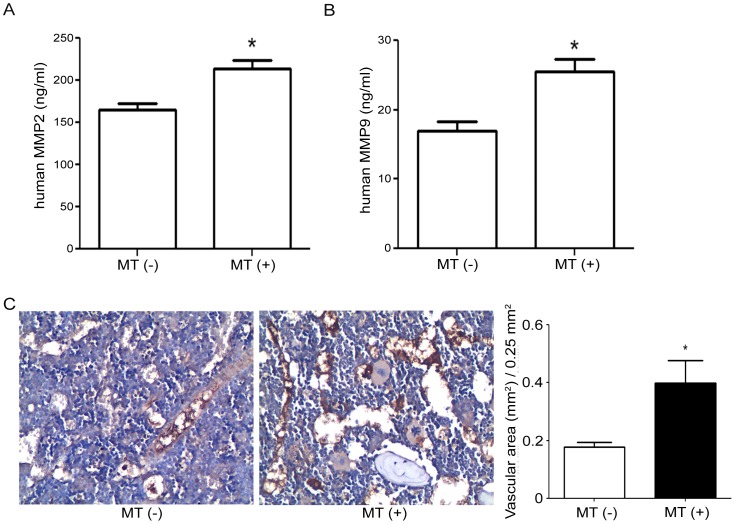
The serum expression level of MMP2 and MMP9 were upregulated while KDR was increased in MT (+). To investigate the possible mechanism of MT induced-metastasis, the serum level of MMP2 and MMP9 was determined. The results showed that after MT treatment, serum level of MMP2 and MMP9 were higher than MT (−) group. MMP2 (A, MT (−) vs MT (+):165±7.8 ng/ml vs 214±9.8 ng/ml, p = 0.0078), MMP9 (B, MT (−) vs MT (+):16.9±1.40 ng/ml vs 25.5±1.81 ng/ml, p = 0.0206). The expression pattern of KDR in MT(+) (0.44±0.077 mm^2^/0.25 mm^2^) was increased than in MT (−) groups (0.18±0.017 mm^2^/0.25 mm^2^) (C).

Under MT treatment, the serum level of MMP2 (214±9.8 ng/ml) was increased compared to MT (−) group (165±7.8 ng/ml) (Fig. 4A, p = 0.0078) and also MMP9 was increased by MT treatment (25.5±1.81 ng/ml) rather than MT (−) group (16.9±1.40 ng/ml) (Fig. 4B, p = 0.0206) indicated that the treatment of MT on OS tumor induced the risk of metastasis through increasing the expression of MMPs. The immunostain of on tumor showed more significant expression pattern of human KDR on tumor vessels in MT (+) group (0.44±0.077 mm^2^/0.25 mm^2^) than in MT (−) groups (0.18±0.017 mm^2^/0.25 mm^2^) (Fig. 4C). The serum expression level of MMP-13, the other MMPs that reported to modulate OS metastasis showed no significance difference between MT (+) and MT (−) groups (figure S1). The outcome suggested the mechanical force potentially induced angiogenesis and indicated the higher risk for tumor metastasis.

## Discussion

Accumulating evidence suggests that manipulation or massage in cancer patient may help to relax the painful symptoms caused by tumor expansion and to ease the stress during therapy[19], [20], [21], [22]. There is also increasing trend that complementary and alternative medicine (CAM) became popular worldwide[23], [24]. Recently, the alternative therapies on cancer care became more popular and acceptable in the world[25], [26] including complementary medicine or supportive therapy such as MT or massage[27]. However, many adverse effects concerning the possibility to promote metastasis have been reported[6], [9], [28]. From clinical findings, many OS patients seek for manipulative therapy to release the uncomfortable symptoms such as swelling or pain before diagnosis without alerting the possibility of OS exist[6]. In our clinical results from 200 OS patients, the metastasis rate were higher and the survival rate were lower in OS patients who had received MT treatment before diagnosis compared with MT (−) patients, respectively (Table 1). Such mechanical massage on cancer patients might lead to tumor cell lesion spreading to lymph nodes near the tumor sites[9], [28] that were reported on breast cancer patients and also proved in OS patients from our clinical and in vivo study. Though MT is a useful supportive therapy for cancer patients after surgery, the treatment should be paid more attention before diagnosis.

Mice osteosarcoma model was established in this study by intratibial injection of human OS cells followed by MT treatment to prove the effects of MT on OS metastasis according to clinical findings. The MT treatment was modified from Bertsch et al[14] and Liu and Huang[29] who claimed that massage promoted cell proliferation or induce gene delivery. Though it was difficult to assay the pressure on mice skin when conducting MT treatment, we standardize the MT protocol by putting the force on xenografted tumor as a touch press pushing back and forth for three times per treatment and two treatments per week for simulating the clinical manipulation protocol. Under MT treatments, the cancer cells migrated not only into bilateral lymph nodes but also in lung tissue that detected by micro-PET/CT and X-ray images on lymph nodes while GFP-labeled cells were noted even in lung (Fig. 2B and 2C, Fig. 3C and 3D). Such GFP-labeled tumor cells have been reported as a strong tool for monitoring the cell metastasis in animals [30], [31]. These results provide direct evidence of MT-induced OS metastasis in vivo. Our results showed obvious cell migration after MT treatment that validated the experiment model. Though tibial injection may cause the leaking of tumor cells, we did not detect any tumor cells in lung of left lymph node in MT (−) group (Table 2) that eliminated the possibility of artificial leaking.

With regard to MT-induced metastasis, there are several mechanisms proposed to explain such phenomenon. 1) mechanical force-induced lymphatic spreading. It was reported that mechanical transport of epithelial cell to axillary lymph node caused by prior surgery manipulation may be through the mechanical force induced cell lesion[9]. 2) mechanical force-induced MMPs expression. Previous studies showed that MMP families were known as a extracellular matrix factor that participates in cell migration that related to tumor metastasis [30], [31]. 3) both mechanisms involved. In OS patients, it was reported that the high expression level of MMPs indicates poor prognosis and higher metastasis risks[32], [33], [34], [35]. The serum level of MMP2 and MMP9 were both up-regulated under MT treatment under the mechanical damage (Fig. 4A and 4B) indicated the highly migration ability of tumor cells while MMP13 also showed the enhanced trend (figure S1). As were reported that massage or MT showed some effects on cancer care that may release pain and improve circulation[36], [37], promoted or stimulated neovessel formation[38], [39], [40], human KDR detected in xenograft injected tumor were highly expressed in MT (+) group compared to MT (−) (Fig. 4C) that supported the higher risk of tumor metastasis and may related to the promotion of blood flow in tumors. Our results did not exclude the possibility of lymphatic spreading or/and increased MMPs expression in MT-induced metastasis. Manipulation or massage therapies are usefully to support and release the tense for clinical care of cancer patients[20], [21], [22], [36]. However, in OS patients, because of the young age that many cases are mis-diagnosed as “growing pain” or “myofacial pain” and search for MT before diagnosed as OS that may influence the prognosis or survival rate. As there are increasing evidence that MT on tumor may take the risk to promote tumor progression and induce metastasis[6], [9], [28] and taken our previous clinical observation and in vivo evidence together, we conclude and suggest that physicians should pay more attention on those patients who seek MT or massage and should take prior diagnosis to get rid of the possibility of osteosarcoma.

## Supporting Information

Figure S1 **The serum expression level of MMP13 showed no significant difference under MT treatment.** The serum MMP13 expression level in MT (+) group (31.4±2.48 pg/ml) was higher than MT (−) (19.1±4.47 pg/ml), with no significant difference (p = 0.07).
(TIF)
