# Supplementary figures and images for: Manipulation Therapy Prior to Diagnosis Induced Primary Osteosarcoma Metastasis—From Clinical to Basic Research
Source: PLoS One. 2014 May 7;9(5):e96571. doi: 10.1371/journal.pone.0096571 (PMC4013034; doi:10.1371/journal.pone.0096571)

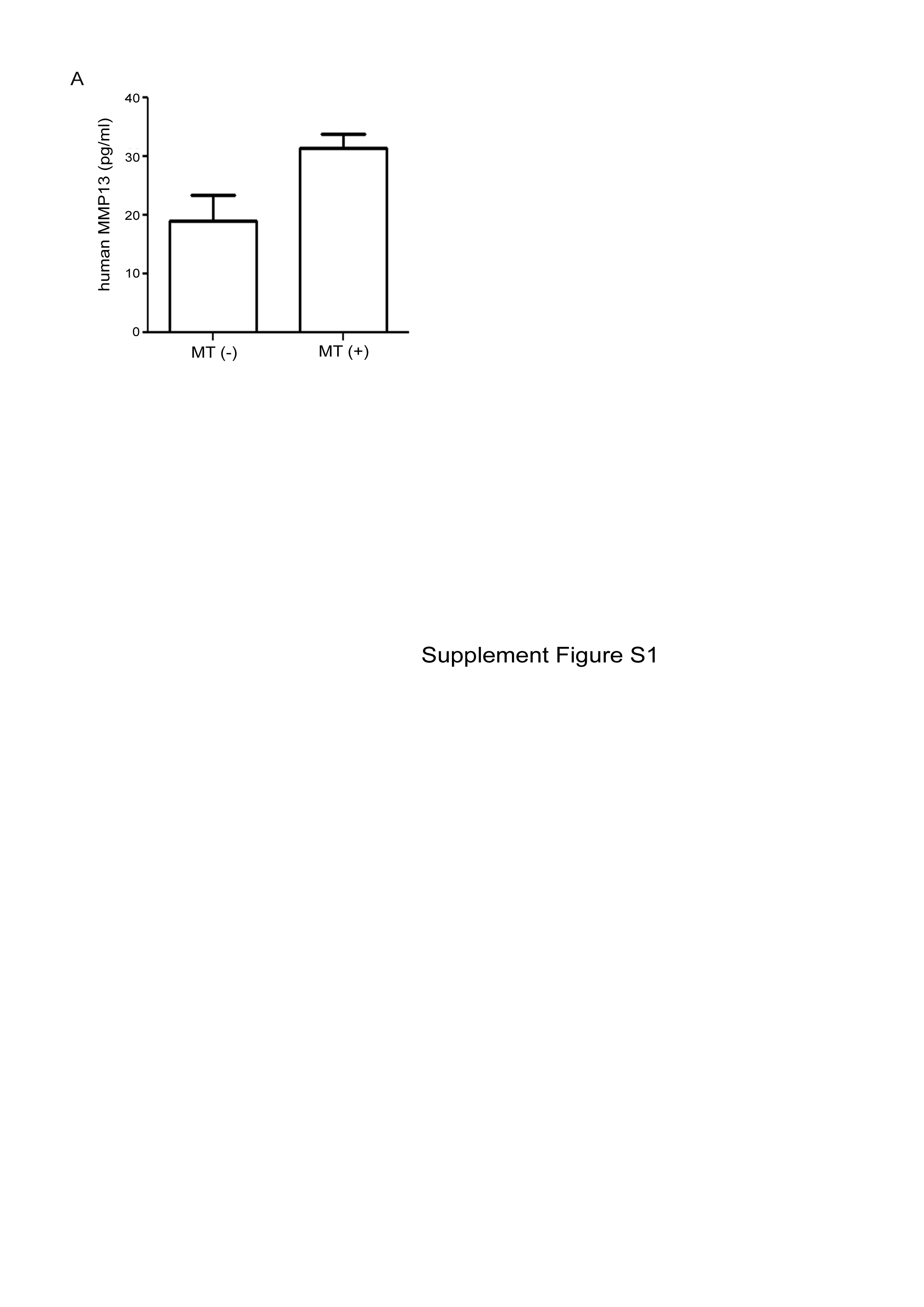

Supplement: Figure S1 — The serum expression level of MMP13 showed no significant difference under MT treatment. The serum MMP13 expression level in MT (+) group (31.4±2.48 pg/ml) was higher than MT (−) (19.1±4.47 pg/ml), with no significant difference (p = 0.07). (TIF) [file pone.0096571.s001.tif]
